# Imaging of the pancreas: state of the art

**DOI:** 10.1186/1470-7330-15-S1-O5

**Published:** 2015-10-02

**Authors:** G Morana, M Fusaro, A Dorigo

**Affiliations:** 1 Radiological Department, General Hospital Ca' Foncello, Treviso, 31100, Italy

## 

Modern imaging of the pancreas has both a morphologic role, with an accurate delineation of the pancreatic tissue as well as of the pancreatic ducts and vessels, and a functional role, with the visualization of the pancreatic fluid cynetic and ductal dilation after secretin injection.

The wide range of informations achievable with modern imaging techniques leads to a in depht knowledge of the different diagnostic value obtained by different imaging techniques.

US is conventionally the first-line diagnostic tool to be used in patients with jaundice or abdominal pain, but with a low sensitivity and specificity, as the imaging feature of a hypoechoic mass cannot provide useful information to differentiate pancreatic cancer from mass-forming pancreatitis. Moreover, artifacts inside cavities can lead to difficulties in differentiating small cystic lesions from solid ones, or a false diagnosis of inhomogeneous content of cystic lesions. With Tissue Harmonic Imaging (THI) a reduction of artefacts inside cystic lesions can be obtained, allowing a better confidence in the nature of small hypoechoic lesions of the pancreas and a better fluid-solid differentiation. Acoustic Radiation Force Impulse (ARFI) technique allows to evaluate the mechanical strain properties of deep organs by means of a focused high intensity pulse to displace the target tissue. It has been utilized in the evaluation of acute pancreatitis, in the differentiation of benign and malignant focal pancreatic solid lesions, in the evaluation of the content of cystic pancreatic lesions [1-3].

Finally, CEUS has improved the characterization of pancreatic tumors, allowing a better differentiation between pancreatic carcinoma and mass-forming pancreatitis, adenocarcinoma and neuroendocrine tumor, serous and mucinous cystic pancreatic lesions [4].

MDCT is a robust technique to explore the pancreas, and it is the gold standard in the local staging of pancreatic carcinoma, thanks to its high spatial resolution, which allows a good depiction of the relationship of the tumor with the peri-pancreatic vessels. However, small iso-attenuating tumors can be hardly depicted with CT. New protocols with low-kV or dual energy technique improve the sensitivity of the MDCT, thus allowing the depiction of small tumors [5,6], especially with the use of new algorithms, which reduce the noise [7].

MRI it is used as a “problem solving” technique, when other imaging modalities are not able to resolve the clinical question, although in the evaluation of cystic tumor of the pancreas it is considered the gold standard, thanks to its high sensitivity to static fluids and different composition of fluids (mucin, blood, etc.). The most significant application of MR in the pancreas are:

- When dealing with Acute Pancreatitis (AP), MR can be the highly accurate in the evaluation of both intra- ed extrapancreatic hemorrhagic/fluid collections.

- MRCP can permit the detection of either acquired (e.g. calculi) or congenital (pancreas divisum) causes of AP, especially after the administration of secretin. This can be particularly useful in case of recurrent AP.

- In Chronic Pancreatic (CP), MRCP enables the distinction between primitive and obstructive causes. Moreover, the functional informations obtained with MRCP after secretin injection is able to suggest the forthcoming onset of a CP when the morphological changes are still not evident.

- In the differential diagnosis between pancreatic carcinoma and mass-forming pancreatitis, morphological, ductal, functional information as well as DWI with IVIM analysis are becoming important tools to achieve a correct diagnosis [8].

- DWI also useful improves diagnostic accuracy for differentiating malignant from benign IPMNs of the pancreas [9].
